# Combination of Tumor Mutational Burden and DNA Damage Repair Gene Mutations with Stromal/Immune Scores Improved Prognosis Stratification in Patients with Lung Adenocarcinoma

**DOI:** 10.1155/2022/6407344

**Published:** 2022-09-20

**Authors:** Dongping Wu, Lingjuan Huang, Jiwei Mao, Jianjiang Liu, Wanli Ye, Jun Xu, Wangyan Zhong, Xiaoyu Zhang, Shenpeng Ying

**Affiliations:** ^1^Department of Radiation Oncology, Shaoxing People's Hospital, Shaoxing Hospital of Zhejiang University, Shaoxing, Zhejiang 312000, China; ^2^Department of General Surgery, Shaoxing People's Hospital, Shaoxing Hospital of Zhejiang University, Shaoxing, Zhejiang 312000, China; ^3^Department of Radiation Oncology, Taizhou Central Hospital, Affiliated Hospital of Taizhou University, Taizhou, Zhejiang 318000, China

## Abstract

**Background:**

Both the tumor environment and the genomic landscape of lung cancer may shape patient responses to treatments, including immunotherapy, but their joint impacts on lung adenocarcinoma (LUAD) prognosis are underexplored.

**Methods:**

RNA sequencing data and whole-exome sequencing results were downloaded from the TCGA database, and only LUAD-related data were included in this study. Based on gene expression data, the ESTIMATE algorithm was used to estimate stromal and immune scores, and CIBERSORT analysis was used for quantification of the relative abundances of immune cells. Somatic mutations were used for calculating tumor mutation burden (TMB). Specific mutations in genes involved in DNA damage repair (DDR) pathways were identified. The individual and joint associations of stromal and immune score, TMB, and DDR gene mutations with 5-year survival were analyzed by the Kaplan–Meier method and multivariate Cox model.

**Results:**

LUAD patients with a high (>highest 25%) stromal or immune score had prolonged survival as compared to those with a low (<lowest 25%) score (log-rank *P*=0.05 and 0.035, respectively). Patients with both high stromal and immune scores had the most favorable survival. Although the survival differences between patients with high (>highest 25%) and low (<lowest 25%) TMB, or between patients with mutant- and wild-type DDR genes were not statistically significant, a survival benefit from high TMB or DDR gene mutations was observed in patients with high stromal or immune scores.

**Conclusion:**

A comprehensive evaluation of transcriptomic signatures and genomic biomarkers may provide a novel avenue for improving prognosis stratification in LUAD.

## 1. Introduction

Lung cancer is the most commonly diagnosed cancer and the leading cause of cancer deaths worldwide [1]. In the past decades, significant advances have been made in the field of lung cancer, including screening for early detection and new agents for survival improvement. The treatment of lung cancer has evolved with the introduction of several lines of tyrosine kinase inhibitors in patients with *EGFR*, *ALK*, *ROS1*, and *NTRK* mutations [2]. More recently, immune checkpoint inhibitors (ICIs), particularly inhibitors of the programmed cell death 1 (PD-1) axis, have dramatically changed the landscape of lung cancer treatment. Clinical trials such as KEYNOTE [3], CheckMate [4], and POPLAR [5] have demonstrated survival benefits for patients with non-small cell lung cancer (NSCLC) who received either immunotherapy, monotherapy, or combined chemoimmunotherapy [2, 6]. Nonetheless, a large portion of NSCLC patients does not respond to ICIs. Even in the large phase III studies that evaluated ICIs combined with chemotherapy for NSCLC patients, overall response rates ranged from 47% to 63% at best [6–9]. Therefore, how to accurately identify a group of patients who will really benefit from immunotherapy poses a consistent clinical challenge. Reportedly, high expression of programmed cell death ligand 1 (PD-L1) in tumor tissues has been associated with a response to ICIs [10]. PD-L1 expression by immunohistochemistry (IHC) testing is an FDA-approved companion diagnostic test for pembrolizumab in NSCLC. However, the discriminatory potential of PD-L1 is doubted due to several limitations, such as intratumor heterogeneity, representativeness of biopsy samples, discordance when defining positive expression, and varied sensitivity of PD-L1 IHC assays [2, 10–13]. In reality, benefits are often seen in patients whose tumors do not express PD-L1, while many patients whose tumors do express PD-L1 expression do not derive benefits from PD-(L)1 blockade [4, 6]. Therefore, identification of high-performance biomarkers associated with response to ICIs and NSCLC prognostication is still an unmet need.

Malignant solid tumor tissues consist of not only tumor cells but also tumor-associated normal epithelial and stromal cells, immune cells, and vascular cells [14]. Stromal cells have important roles in tumor growth, disease progression, and drug resistance [14]. Generally, tumor tissue shapes the immune suppressive microenvironment to prevent *T* cells from infiltrating the tumor site, and enhancing *T* cell infiltration can improve cancer immunotherapy [15–17]. For example, an increase of CD8^+^tumor-infiltrating lymphocytes (TILs) has been reported to be associated with increased sensitivity to ICIs and favorable prognosis [15–17]. Yoshihara et al. recently developed a novel method, called ESTIMATE (estimation of stromal and immune cells in malignant tumor tissues using expression data), to infer the fraction of stromal and immune cells in tumor samples using gene expression signatures [14]. The potential of stromal and immune scores for predicting prognosis and response to immunotherapy has been suggested by recent studies conducted in patients with solid tumors, such as gastric [18], liver [19], renal [20], head, neck [21], and lung [22–27] cancers. In addition, more investigations have focused on the identification of predictive biomarkers for response to ICIs in lung adenocarcinoma (LUSD) patients, which may identify potential beneficiaries to guide clinic treatment. For example, LUSD patients with high levels of B2M protein were detected by more *T* and natural killer cells in their tumors and associated with an increased response to PD-1-based immunotherapy [28]. *VTCN1* (*B7*-*H4* gene), which negatively associates with granzyme B levels, contributes to immunosuppression for LUAD patients harboring EGFR-activating mutations [29]. Somatic copy number alterations (SCNAs) burden is negatively associated with ICIs progression-free survival [30]. In addition to gene expression signatures or SCNAs-based biomarkers, tumor mutation burden (TMB) has arisen as another potential indicator of response to ICIs, with the premise that an increase in TMB leads to an increased number of mutated proteins, or neoantigens, on the surface of tumor cells capable of eliciting an immune response [2, 6]. In addition, an association between mutation burden and sensitivity to ICIs is also evident in the hypermutated tumors of patients with deleterious alterations in DNA-repair genes such as *MLH1*, *MSH2*, *MSH6*, and *PMS2*, which are characterized by increased CD8^+^*T*-cell infiltrates, as well as malignancies with mutations in *BRCA2, POLD1,* and *POLE* [6]. Although several recent studies reported the prognostic value of stromal and immune scores in LUAD, the predominant histological subtype of NSCLC, by analyzing data from the Cancer Genome Atlas (TCGA) and/or the Gene Expression Omnibus (GEO) projects [22, 23, 25–27], none of these studies put both gene expression and mutation data in the same analytical framework.

In the current study, based on TCGA RNA sequencing data and whole-exome sequencing (WES) data, overall survival (OS) benefits were identified in LUAD patients with either a high stromal/immune score alone or in a combination of high TMB or mutant genes in DNA damage repair (DDR) pathways. Because the TCGA database lacks detailed information about treatments, including immunotherapy, the analyses in this study focused on assessing the prognosis stratification effects of these potential biomarkers.

## 2. Materials and Methods

### 2.1. Data Source

Data on gene expression and genomic variants, as well as clinicopathological information, were obtained from the TCGA database (https://tcga-data.nci.nih.gov/tcga/), which was jointly created by the National Cancer Institute and the National Human Genome Research Institute in 2006. It offers a comprehensive catalog of genomic, epigenomic, transcriptomic, and proteomic alterations that occur in 33 major cancer types [31]. Only LUAD-related data were extracted in this study. In brief, RNA sequencing data of tumor tissues were downloaded from 513 LUAD patients, and among them, 505 had clinical data. The TCGA WES data from 565 LUAD patients were used for mutation calling, and 479 of these patients also had gene expression and clinical data. After excluding 11 patients without information on time to death/last follow-up for survival analysis, the final dataset included 468 treatment-naïve LUAD patients.

### 2.2. Estimation of Stromal and Immune Scores

The ESTIMATE algorithm (*R* package “estimate”) was used to estimate the stromal and immune scores from the TCGA gene expression data [14]. The estimations were based on 141 stromal genes and other 141 different genes related to immune cell infiltration [14]. An analytical tool, named CIBERSORT (cibersort.stanford.edu), which characterizes the cell composition of complex tissues from their gene expression profiles [32], was used to quantify the relative abundances of 22 types of immune cells. LUAD patients were divided into high stromal/immune score (upper quartile) and low stromal/immune score groups (lower quartile).

### 2.3. Mutation Identification and TMB Quantification

The Mutect2 results from the TCGA WES data were used for identifying somatic mutations. The TMB of a tumor sample is calculated by the number of nonsynonymous somatic mutations (single nucleotide variants and small insertions/deletions) per megabase in coding regions [33, 34]. Moreover, stratified analyses according to mutant- and wild-type genes were conducted for those related to DDR pathways, including base excision repair (BER: *POLE, MUTYH*), checkpoint factors (*ATM, ATR, CHEK1, CHEK2*), Fanconi anemia (FA: *BRCA2, BRIP1, FANCA, FANCC, FANCD2, PALB2, BLM*), homologous recombination repair (HRR: *BRCA1, MRE11A, RAD50, RAD51*), and mismatch repair (MMR: *MLH1, MSH2, MSH6, PMS2*) [35].

### 2.4. Statistical Analysis

**Figure 1:**
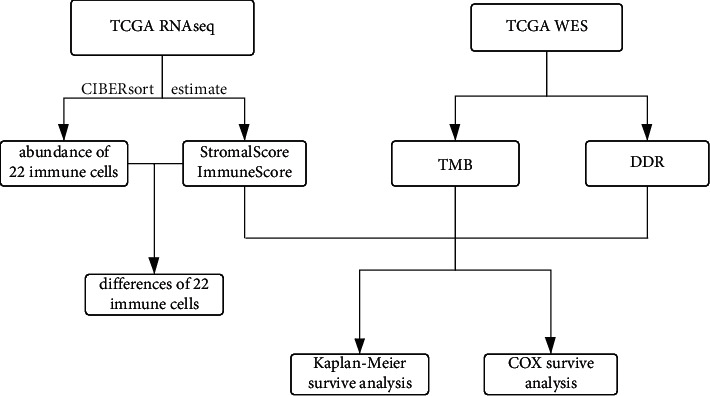
Flow chart of analytic procedure.

The distributions of stromal and immune scores were visualized using violin plots. A Wilcoxon test was used to compare the differences in medians between two comparison groups. OS was the clinical endpoint analyzed in this study. Survival curves were plotted using the Kaplan–Meier method, and survival differences were compared by a log-rank test [36]. The associations between prognostic factors and OS in LUAD patients were evaluated using the multivariate Cox model [37]. A detailed analytic procedure is shown in Figure 1. Statistical analyses were conducted using *R* software (version 3.6.1, packages “ggplot2,” “survival,” and “survminer”) [38, 39]. A *P* value of 0.05 (two-sided) was set as the cutoff point of statistical significance.

## 3. Results

### 3.1. Distribution of Stromal and Immune Scores

**Figure 2:**
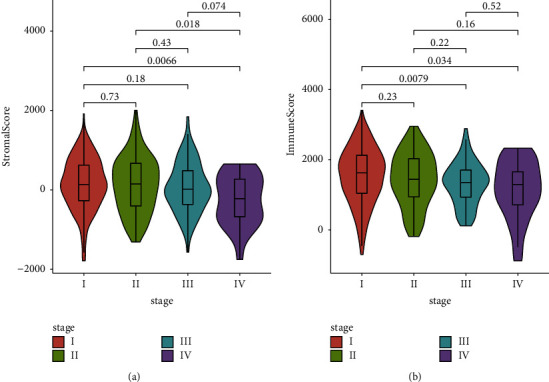
Violin plots to show the distributions of (a) stromal scores and (b) immune scores across tumor stages. The differences in medians between the two comparison groups were compared by the Wilcoxon test and *P* values were presented to indicate statistical significance. ^*∗*^*P* ≤ 0.05 and ^*∗∗*^*P* ≤ 0.01.

**Figure 3:**
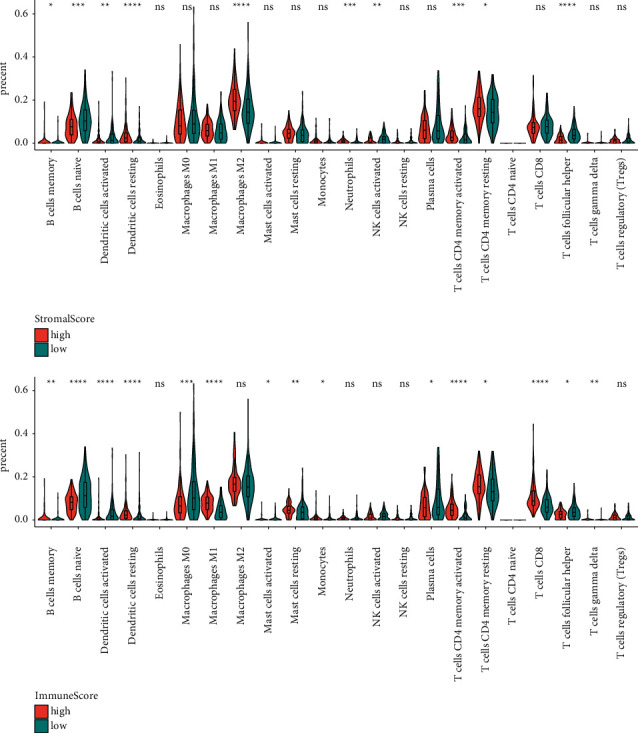
Relative abundances of immune cells in patients with high or low stromal/immune scores. ^*∗*^*P* ≤ 0.05, ^*∗∗*^*P* ≤ 0.01, ^*∗∗∗*^*P* ≤ 0.001, and ^*∗∗∗∗*^*P* ≤ 0.0001.

Among the 468 LUAD patients included in the analysis, 216 (46.2%) were males, 399 (85.3%) had a smoking history, and 114 of them were current smokers. There were 259, 108, 77, and 24 patients with stage I, II, III, and IV LUAD, respectively. The stromal and immune scores for each patient were estimated using the ESTIMATE algorithm. The score distributions according to different tumor stages are visualized in Figure 2. Patients with stage IV tumors had significantly lower stromal scores than those with either stage I or stage II tumors (*P*=0.007, 0.018, respectively). Similarly, patients with stage I tumors had significantly higher immune scores than those with advanced-stage tumors (*P*=0.008 when compared with stage III tumors and *P*=0.034 when compared with stage IV tumors). Furthermore, the relative abundances of 22 types of immune cells were quantified using CIBERsort software.  Figure 3 depicts the abundances of each kind of immune cell between high or low stromal/immune score groups. Significant differences were observed in the abundances of *T* cells, *B* cells, and macrophages when comparing the high-score group with the low-score group. Specifically, there was more CD8+ *T* cell infiltration in the patients with a high immune score.

### 3.2. Survival Analysis Based on Stromal and Immune Scores

**Figure 4:**
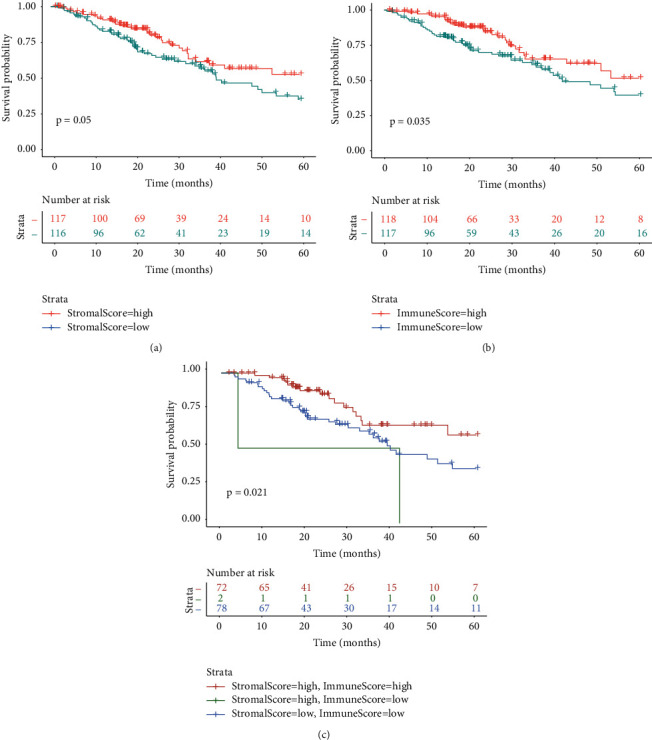
Kaplan–Meier survival curves to show differences in the 5-year survival (a) between patients with high and low stromal scores, (b) between patients with high and low immune scores, or (c) among different comparison groups which were defined according to both stromal and immune scores.

Patients were divided into three groups based on their stromal or immune score quartiles: high-score group with a score in the 4^th^ quartile (>highest 25%), low-score group with a score in the 1^st^ quartile (<lowest 25%), and medium-score group with a score in the 2^nd^ or 3^rd^ quartiles. The 5-year survival curves were plotted using the Kaplan–Meier method. As shown in Figure 4, LUAD patients with either a high stromal score or a high immune score had a prolonged survival as compared to those with a low score (log-rank *P*=0.05 and 0.035, respectively, in Figures 4(a) and 4(b)). To illuminate the joint effect of stromal and immune scores, the patients were further stratified into four groups: patients with both high stromal and immune scores (*N* = 72), patients with a high stromal score and a low immune score (*N* = 2), patients with a low stromal score and a high immune score (*N* = 0), and patients with both low stromal and immune scores (*N* = 78).  Figure 4(c) shows that patients with both high stromal and immune scores had the most favorable survival.

### 3.3. Survival Analysis in Combination with TMB

**Figure 5:**
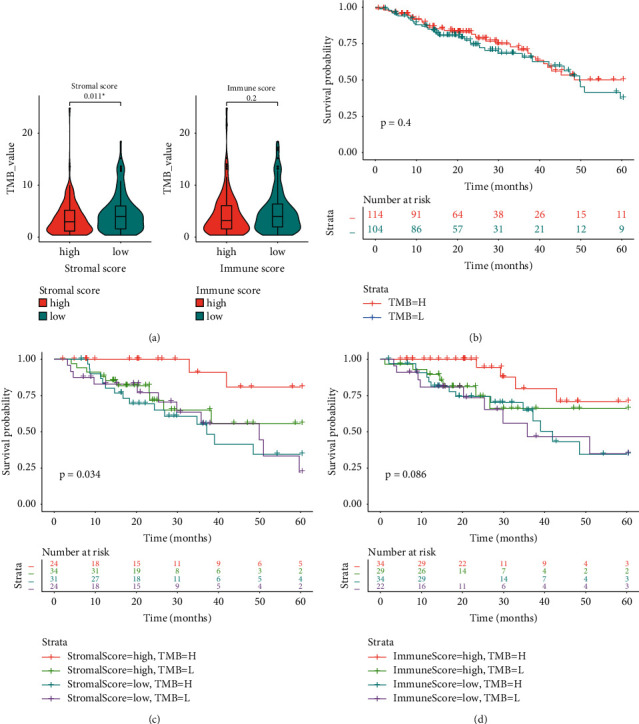
Integrative analyses of tumor mutation burden (TMB) and stromal/immune scores. (a) TMB levels in patients with high and low stromal/immune scores; ^*∗*^*P* ≤ 0.05. (b) The survival difference between patients with high and low levels of TMB. (c) The survival difference among four groups of patients classified according to the level of TMB and stromal score. (d) The survival difference among four groups of patients stratified according to the levels of TMB and immune score.

TMB was calculated on the basis of the Mutect 2 results from TCGA available WES data of LUAD. As shown in Figure 5(a), a slightly higher TMB was detected in patients with low stromal scores than in those with high stromal scores (*P*=0.011), but the TMB difference between patients with high and low immune scores was not statistically significant. LUAD patients were then stratified into high-TMB (>highest 25%), medium-TMB, and low-TMB (<lowest 25%) groups according to their TMB levels. The 5-year survival was similar between patients with high and low levels of TMB (log-rank *P*=0.40, Figure 5(b)). A comprehensive analysis was conducted by incorporating TMB levels into stromal or immune score-based analyses, similar to the patient stratification in the joint analyses of stromal and immune scores. Figures 5(c) and 5(d) indicate that OS benefits from high TMB were only observed in those with high stromal or immune scores.

### 3.4. Survival Analysis in Combination with DDR Gene Mutations

**Figure 6:**
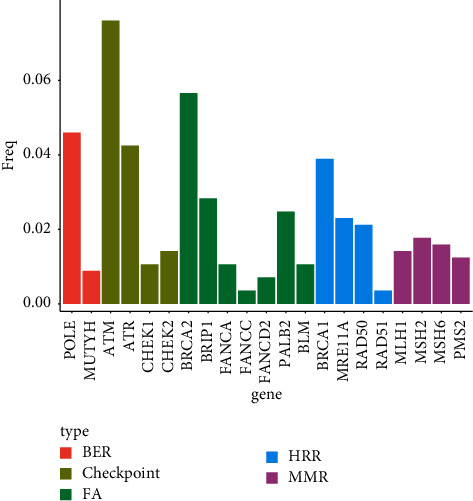
Frequencies of mutant genes involved in DDR pathways.

**Figure 7:**
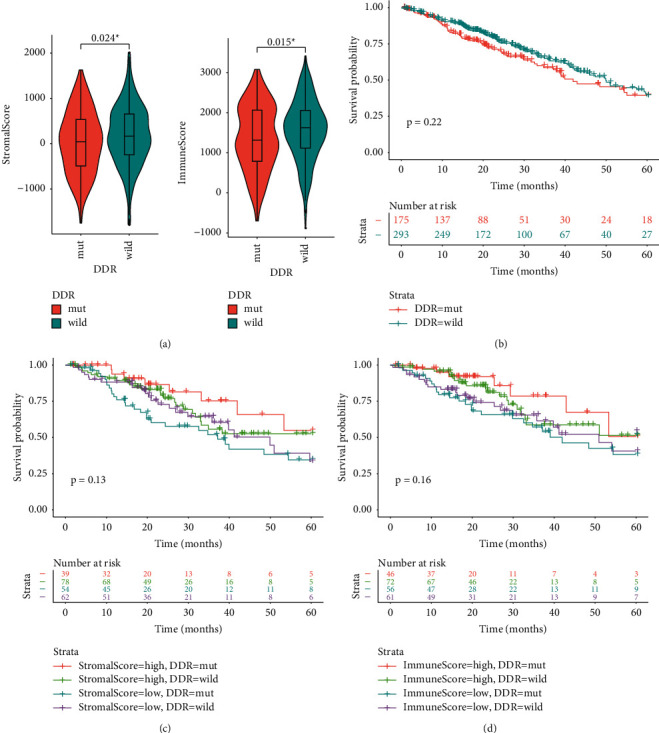
Integrative analyses of mutations in DNA damage repair (DDR) genes and stromal/immune scores. (a) Stromal and immune scores in patients with mutant- or wild-type DDR genes. (b) The survival difference between patients with mutant- and wild-type DDR genes; ^*∗*^*P* ≤ 0.05. (c) The survival difference among four groups of patients classified according to the status of DDR genes and stromal score. (d) The survival difference among four groups of patients stratified according to the status of DDR genes and immune score.

Twenty-one genes involved in DDR pathway were analyzed. As shown in Figure 6, the top three mutant genes in these LUAD samples were *ATM* (7.6%), *BRCA2* (5.7%), and *POLE* (4.6%). Patients with mutant DDR genes had significantly smaller stromal and immune scores than those with wild-type genes (*P*=0.024 and 0.015, respectively, Figure 7(a)). Although patients with mutant-type DDR genes had a relatively lower survival, the survival difference between those with mutant- and wild-type genes did not achieve statistical significance (log-rank *P*=0.22, Figure 7(b)). When conducting survival analyses by incorporating the mutation status of DDR genes into stromal and immune scores, the improved survival was dominated in patients with both high stromal/immune scores and mutant-type DDR genes (Figures 7(c) and 7(d)).

### 3.5. Multivariate Analysis in Combination of Stromal/Immune Scores, TMB, and DDR Gene Mutations

**Figure 8:**
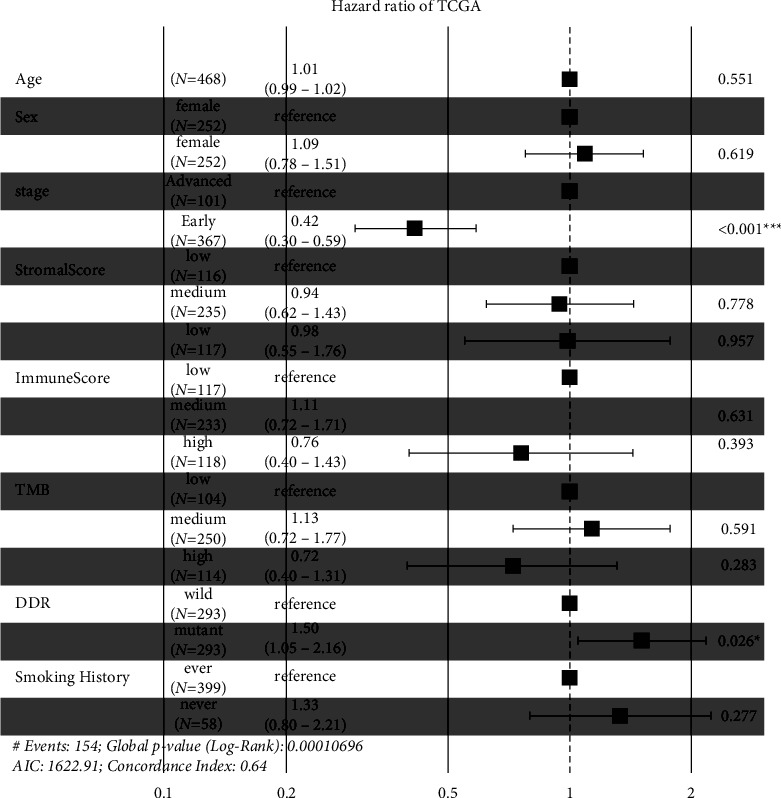
Multivariate Cox analyses in combination of stromal/immune scores, TMB, DDR gene mutations, and clinicopathological variables. TMB, tumor mutation burden; DDR, DNA damage repair; HR, hazard ratio; CI, confidence interval.

A multivariate Cox model including age, sex, smoking status, tumor stage, stromal score, immune score, TMB, and mutation status of the DDR gene was developed to evaluate the independent predictive value of these biomarkers. As shown in Figure 8, compared with late-stage patients, early-stage patients had a 58% (hazard ratio [HR] 0.42, *P* < 0.001) decreased risk of death. The death risk increased by 50% (HR 1.50, *P*=0.026) among patients with mutant-type DDR genes in comparison to those with wild-type genes. Although patients with a high immune score or a high TMB level had a lower risk of death, the associations were not independent of covariates.

## 4. Discussion

Over the past several years, ICIs, which target inhibitory receptors on *T* cells and reinvigorate antitumor immune responses, have begun to transform clinical cancer care [10]. Because only a subset of patients derives clinical benefit from ICIs, it is critical to identify a specific biomarker or a group of biomarkers with high performance in discriminating potential responders from nonresponders. The determination of tumor PD-L1 expression by IHC has been extensively studied as a predictor of response to anti-PD1/PD-L1 agents, although it is sometimes inconclusive. Expression of PD-L1 on infiltrating immune cells in the tumor microenvironment has also been associated with clinical response to immunotherapy in cancers, including NSCLC [10, 15]. In particular, it has been recently mentioned that the combined use of the TTF1/PD-L1 score outperforms the gold standard PD-L1 biomarker for OS prediction in LUSD [40]. The characterization of the tumor microenvironment and its interaction with host genomics will help to optimize precision medicine and prognosis management. In this study, by analyzing data from the TCGA, the associations of gene-expression-based biomarkers and genomic-variant-based biomarkers with 5-year survival were evaluated individually and jointly. Significantly improved survival was observed in patients with high stromal and/or immune scores. Although using TMB level or DDR gene mutations alone could not differentiate patient risk for death, a combination of these biomarkers with stromal/immune scores has the potential to identify patients with OS benefits.

The tumor microenvironment consists of factors extrinsic to cancer cells, including various immune and stromal cells, vasculature, extracellular matrix, and cytokines that influence response to therapy [41]. The crosstalk between cancer cells and tumor stroma plays an important role in the progression of tumors and their metastasis [42]. The density of TILs in the tumor microenvironment confers a prognostic and predictive impact on some tumor types, including NSCLC, regardless of ICI therapy [10, 43, 44]. A metric known as the Immunescore, which involves quantification of CD8+ *T* cells at the center and periphery of a tumor, was reported to be a strong predictor of OS that can complement traditional TNM staging or microsatellite instability (MSI) status in colorectal cancer [45–47]. The *T*-cell-inflamed gene expression profile and immune gene expression signatures also represent emerging predictive biomarkers [48]. Stromal and immune scores are stromal tissue and ICIs related gene expression signature-based biomarkers recently developed by Yoshihara et al. [14] and have been associated with prognosis in lung cancer patients [22–27]. In this study, high stromal and immune scores, analyzed individually or jointly, were associated with improved 5-year survival, which was consistent with previous findings derived from the TCGA LUAD dataset [22, 25–27]. Moreover, CIBERSORT analysis identified more CD8+ *T* cells in the patients with a high immune score, highlighting the importance of the TIL phenotype in LUAD prognosis.

Cancer is a genetic disease, and neoplastic transformation results from the accumulation of somatic mutations in the DNA of affected cells [48]. Considering that high TMB correlates with a greater probability of displaying tumor neoantigens on human leukocyte antigen molecules on the surface of tumor cells, it is rational to hypothesize that the tumors with the highest TMB are more likely to respond to ICIs because greater mutation load would increase the likelihood of recognition by neoantigen-reactive*T* cells [48]. Consistent with this hypothesis, several studies have demonstrated an association between high TMB and a response to ICIs in NSCLC [49–51]. Pembrolizumab, an anti-PD1 agent, was recently FDA-approved in TMB-high advanced solid cancers in response to results from the KEYNORE-158 trial [52]. Nevertheless, in the complex multiarm CheckMate227 trial in NSCLC, neither TMB nor PD-L1 expression could segregate therapy responsiveness [53]. In the present study, patients with high TMB had a slightly prolonged survival, but the difference in OS between patients with high and low levels of TMB was not statistically significant. This result was consistent with data from previous studies that demonstrated clinical benefit from high TMB with respect to objective response rate or progression-free survival, rather than OS [48]. The reason for not achieving OS improvement may be attributed to the fact that tumors with increased mutations and genomic instability can adapt more quickly to immune pressure, resulting in treatment resistance [6]. Furthermore, in this study, the survival advantage from high TMB was only observed in patients with high stromal or immune scores, suggesting an approach to improve prognosis stratification by using a biomarker panel in combination of gene expression and genomic variants.

In addition to the overall mutation burden, TMB-related specific mutations such as *MGA* [54], *EPHA5*, [55], *CTNNA2* [56], and co-mutation of *FAT3* and *LRP1B* [57] have been found as novel predictive biomarkers for ICIs response in nonsquamous NSCLC or LUSD. Hypermutation of driver genes may have a distinct impact on MSI in tumors [58]. The deficiency in DDR genes such as *MLH1, MSH2, MSH6*, *PMS2,* and *POLE* has high concordance to high MSI (MSI-H), and the concordance of MSI and MMR testing in prior studies was reported to be 92% [59, 60]. In the study of Rizvi, mutations of genes involved in the DDR pathways were enriched in patients who derived clinical benefits from anti-PD1 therapy [49]. A recent large study reported that, in MSI-H cases, the presence of DDR alterations correlated with a significantly higher TMB as compared with DDR-wild-type MSI-H cases [61]. Therefore, in this study, it was not surprising to observe an OS improvement in the patients with both high stromal/immune scores and mutant DDR genes, similar to the combined analysis with TMB. In addition, although DDR gene mutation was shown to be an independent prognostic factor in the multivariate analyses, due to the retrospective nature of this study, it is unclear whether the DDR alteration is causative of the higher TMB or a result of the high TMB phenotype, thus further investigations are needed for exploration.

This study comprehensively evaluated the prognostic values of transcriptomic signatures and genomic biomarkers and provided a novel avenue for improving prognosis stratification in LUAD. However, the current study has several limitations. First, the TCGA database lacks information on detailed treatments, so stratified analyses according to a given treatment such as immunotherapy are infeasible. Second, the biomarkers identified in this study could not be compared with other wildly used biomarkers such as tumor and immune cell PD-L1 expression due to the unavailability of these data. Third, in the combined analyses of stromal/immune scores, TMB, and DDR gene mutations, the statistical power may be insufficient due to a small number of patients in each subgroup. Fourth, the relative abundances of 22 types of immune cells were estimated using a bioinformatic tool, which may differ from the real situation in tumors. Finally, this was a retrospective analysis based on a publicly available database and no independent validation was conducted to further test the potential of these biomarkers. Large prospective studies with completed treatment-related data are warranted to confirm the findings in this study.

## Data Availability

Publicly available datasets were analyzed in this study. All LUSD relevant RNA sequencing and whole-exome sequencing data can be downloaded from the TCGA database (https://tcga-data.nci.nih.gov/tcga/).
